# Does the Fatigue Induced by a 30-Minute Run Affect the Lower Limb Acceleration Spikes’ Asymmetries?

**DOI:** 10.3390/bioengineering12030294

**Published:** 2025-03-14

**Authors:** Gabriel Delgado-García, Isabel M. Martín-López, Fulgencio Soto-Méndez, Arturo Quílez-Maimón, Salvador Boned-Gómez

**Affiliations:** SER Research Group, Physical Activity and Sport Sciences Department, CESAG, Comillas Pontifical University, 07013 Palma de Mallorca, Spain; immartin@cesag.comillas.edu (I.M.M.-L.); fsoto@cesag.org (F.S.-M.); aquilez@cesag.org (A.Q.-M.); sboned@cesag.org (S.B.-G.)

**Keywords:** articular damage, biomechanical loading, disbalances, exertion, laterality, locomotion, musculoskeletal injuries, overuse injury, runners, shock attenuation

## Abstract

Running-induced fatigue affects several biomechanical parameters, and yet few studies are focused on the acceleration spikes’ asymmetries. This study aimed to evaluate the effects of a 30 min run on lower limbs spikes’ asymmetries. Eighteen recreational runners (35.6 ± 7.5 years; seven women) performed a treadmill running protocol at a moderate speed and acceleration spikes’ asymmetries and kinematic (temporal) parameters were measured via accelerometers—on the tibias and sacrum—and photogrammetry. Acceleration spikes’ parameters were continuously measured and averaged per minute to assess the relationship between fatigue and acceleration spike asymmetries via a linear regression model. Right tibial acceleration spikes increased over time (r = 0.9; *p* < 0.001) and left tibia spikes decreased (r = 0.78; *p* < 0.001), with a rise in tibial load asymmetry from 9% to 25% at the end (r = 0.98; *p* < 0.001). This study suggest that fatigue affects the acceleration spikes of the two legs differently, with increasingly greater acceleration spikes in the right (dominant) leg. These findings should be considered, as greater asymmetries are related to overuse injuries and lower efficiency. Also, in studies focusing on running mechanics with fatigue, it is recommended that researchers collect data from both limbs, and not only from the right (dominant) leg.

## 1. Introduction

The human body exhibits asymmetry due to both genetic and environmental factors [1]. These asymmetries can manifest at both functional [2] and structural levels [3]. In locomotion, a functional asymmetry exists between the two legs, with the dominant limb contributing more to propulsion, while the non-dominant limb plays a greater role in stabilization or braking [2]. However, excessive kinetic and kinematic asymmetries in running have been associated with an increased risk of injury [4,5] and lower metabolic efficiency [6]. Furthermore, fatigue generally impairs running technique, both in kinematics and kinetic terms. For instance, fatigue induces alterations in spatiotemporal variables such as contact time, flight times, and cadence [7,8,9,10,11] or kinetic variables such as ground reaction forces [12,13,14]. Although previous researchers have theorized that fatigue will increase the occurrence of asymmetry [5], recent research has yielded contradictory results [15,16]. Several authors have found no relationship between asymmetries and fatigue generated by running or other protocols of a different nature [3,14,16,17,18,19,20,21,22,23]. However, some findings suggest that fatigue exacerbates asymmetries [13,15,24]. Conversely, one study found that fatigue induced a decrease in asymmetries, which could be due to a motor strategy to compensate for the mechanical load on both legs [25]. These discrepancies may be due to the fatigue protocols used, the level of the runners, or the parameters used to assess asymmetries.

Regarding the parameters used to evaluate changes in asymmetries with fatigue, most research analyzes kinetic and kinematic variables by combining 3D motion capture systems with force platforms typically integrated into force-instrumented treadmills. Few use accelerometers, though this is a technology widely used to measure mechanical loading in the lower limbs [26,27,28,29], which has been associated with joint and cartilage degeneration [30,31,32]. According to our literature review, no study to date has analyzed the impact of fatigue on asymmetries of the acceleration spikes measured with accelerometers. From a technical perspective, accelerometers enable the continuous measurement of acceleration spike asymmetries during the test, even at high sampling rates, accounting for acceleration spikes at each step. Furthermore, accelerometers show a good reliability for measuring shank acceleration spikes across short- and long-term running time periods [27]. Additionally, when placed at different caudocranial locations, accelerometers allow for the estimation of acceleration spike attenuation during running. In this way, it has been found that acceleration spikes are greater in the lower limbs than in the spine and head and that this variable could be related to injuries due to overuse [27,28,29].

Hence, the main aim of this study was to determine if the fatigue induced by a running test at a constant speed is related with the lower limbs’ asymmetries in the acceleration spikes, representing a novel approach. A secondary objective was to analyze the effects of fatigue on the acceleration spikes (i.e., magnitude and attenuation) and temporal variables (flight, contact times, and cadence). It was hypothesized that fatigue would impair running technique, generating an increase in asymmetries and an increase in the acceleration spikes and contact times. The finding of this study improve our understanding of how fatigue affects kinematics, acceleration spikes and their asymmetries, variables related to metabolic efficiency, and the risk of overuse injury. Moreover, fatigue’s demonstrated influence on asymmetries reinforces the importance of assessing both lower limbs in studies in which fatigue is generated, and not only the dominant limb, as is frequently the case.

## 2. Materials and Methods

### 2.1. Sample

Eighteen runners (35.6 ± 7.5 years; seven women) participated in this study, who volunteered in response to an advertisement, after being duly informed about our research. Their mean time in a 10 km test was 43.21 ± 20.68 min. The dominant lower limb was determined from the runners’ verbal response on which limb they would use to kick a soccer ball [3]. All runners reported right-leg dominance. Anthropometric and cardiovascular data of the subjects are shown in Table 1. All runners completed a questionnaire addressing injuries sustained in the preceding months, medical conditions that could influence study outcomes or pose health risks, running experience, weekly training volume, and personal best performances in official 10 km and half-marathon races. The inclusion criteria for participation in the study were (1) amateur runners under 50 years of age who were in training and (2) had participated in a 10 km race in the last 12 months. Subjects were excluded if they (1) had thyroid dysfunction, infections, or chronic diseases; (2) had suffered an ischemic vascular event in the past months; (3) abused alcohol; (4) had an injury that prevented them from performing the study tests; (5) were unwilling or unable to comply with the procedures; or (6) did not meet other criteria of the investigators.

Runners were instructed to abstain from any strenuous physical activity and consumption of stimulants (e.g., coffee) 48 h before the test.

Data were processed in accordance with national data protection protocols related to personal details. This study was conducted in accordance with ethical principles for medical research involving human subjects and ethical standards of the Declaration of Helsinki. It also received a favorable evaluation from the Bioethics Committee of Comillas Pontifical University.

### 2.2. Procedures and Instruments

Runners were asked to perform two treadmill (NordicTrack X7i, NordicTrack Inc., Logan, UT, USA) running tests on different days (with a minimum and a maximum of 7–15 days difference): an incremental test to determine the so-called fatigue speed and a 30 min fatigue test, performed at the fatigue speed pace.

On both days, runner height (SECA tallimeter, Hamburg, Germany), weight/body composition (Tanita BC545N, Tanita, Tokyo, Japan), and blood pressure (Omron M6 Comfort, Omron Healthcare, Kyoto, Japan) were measured. The room temperature was set at 20 degrees using an air conditioner (Mitsubishi electric MSZ, Mitsubishi, Tokyo, Japan) and temperature and humidity were also measured (via an Extech 445814 hygrometer-thermometer, Tokyo, Japan). The slope of the treadmill incline was set at 1%, as this slope has been shown to accurately reflect the energy cost of outdoor running [24].

On the first day, runners performed the incremental test. The test began with an 8 min warm-up at a self-selected speed between 8 and 9 km/h. Following the warm-up, the test was started at 8 km/h and every minute the speed was increased by 0.5 km/h until the maximum speed was reached [33]. The test was stopped when the subjects could not maintain the imposed pace or showed clear signs of exhaustion.

On the second day, the runners completed a 30 min fatigue test, designed to address the primary aim of the research. The test begins with an 8 min warm-up at the same self-selected speed (8–9 km/h) used in the incremental test. After the warm-up, the 30 min fatigue test was started at a speed corresponding to 80% of the speed of the last completed sector in the incremental test (fatigue speed). This approach was adapted from Strohrmann et al. [34], who used a running speed of 85% of the velocity attained in an incremental test for a 45 min fatigue protocol. Based on pilot trials and the perceived fatigue levels reported by multiple runners, we opted to set the running speed at 80% of the velocity reached in the final stage of the incremental test. This adjustment ensured that nearly all participants could complete the fatigue test. The duration of the running protocol in the present study (30 min) was similar to other studies examining the effects of fatigue on kinematics or kinetics [3,16,32,35].

During both tests, the heart rate was continuously monitored and averaged per minute to quantify the fatigue, using a pulsometer Polar H10 (Polar Electro Oy, Kempele, Finland). Runners wore their own running shoes (including any foot orthosis), which were the same in both tests, ensuring consistency across tests and minimizing alterations to their natural running conditions.

#### 2.2.1. Acceleration Spikes’ Asymmetry Analysis

**Figure 1:**
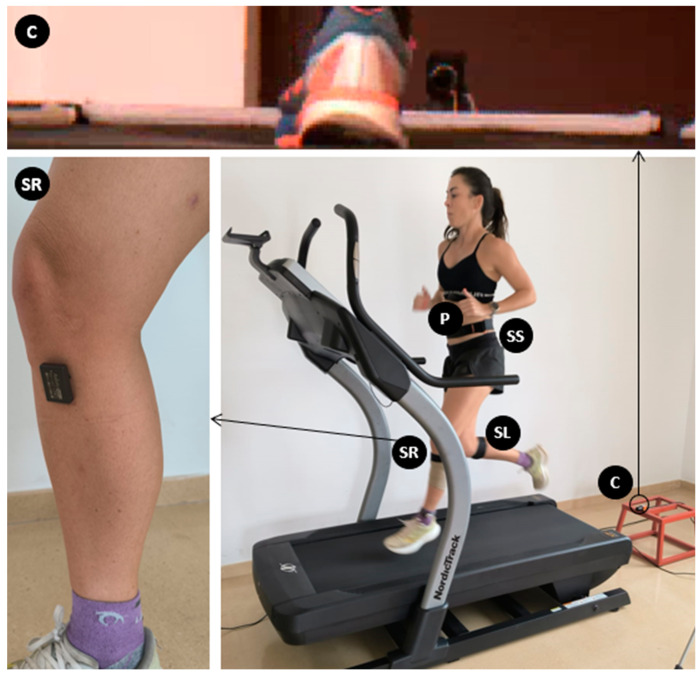
Experimental setup: high-speed camera (C) to determine contact and flight times; placement of the inertial sensors (right tibia sensor [SR], left tibia sensor [SL], and sacrum sensor [SS]) and pulsometer (P). The image on the left shows the exact location of the tibia sensors (the elastic band has not been placed on top to show the exact location). The photo above shows a screenshot from the high-speed camera video.

Acceleration spikes were measured based on the magnitude of the acceleration signal recorded by inertial sensors [28]. Three Axivity AX6 inertial sensors (Axivity, York, UK) with a measurement range of ±16 Gs and with a sampling frequency of 400 Hz were used. Two of them were placed under the medial epicondyle of the right and left tibias, with the longitudinal axis aligned to the tibia, to analyze the acceleration spike on the knees (Figure 1). The third sensor was placed over the L5 vertebra with the longitudinal axis aligned with the vertical, to analyze the acceleration spike on the sacral region. A 3M adhesive tape (3M Health Care, St Paul, MN, USA), which wrapped around and adhered directly to the skin, and an external neoprene elastic belt (Xsens), were used to secure the sensors. The Axivity sensor data were imported into OriginLab (Northampton, MA, USA), and the negative spikes on the x-axis of the sensor (the highest spikes) were selected without applying any filter, to obtain a more accurate representation of the variability within the system than if a filter was applied [36]. This was carried out using the specific tool “Find Peaks” of Originlab [28]. Based on pilot trials, the tool was configured for the selection of local maxima in windows of 75 samples in the case of the sensor placed in the sacral region and 150 samples in the sensors placed on the tibias. Subsequently, a visual check of the selection of the spikes was carried out, to ensure that no spikes were missed or that no unwanted spikes were selected.

In order to compute the attenuation between the tibia and sacrum acceleration spike magnitude, the sacral acceleration spike corresponding to each right and left step was chosen. This was achieved using a custom-built Excel template designed to select the adjacent sacral acceleration spike nearest to each tibial acceleration spike [28]. The Excel template with a real example is provided within the Supplementary Materials. Then, attenuation of the acceleration spikes between the tibia and the sacrum region was calculated by subtracting the magnitude of the acceleration spike of the tibia from the magnitude of the adjacent acceleration spike on the sacrum region, dividing the result by the mean of these two variables, and multiplying it by 100 [28,29]. A positive value denotes a higher acceleration spike in the tibia than in the sacrum region and vice versa.

The time in milliseconds between the tibia and sacrum acceleration spikes (tibia-to-sacrum spike delay) was taken into account to ensure that they were close in time and therefore generated with the same step (right or left) [28]. Additionally, the time interval between consecutive acceleration spikes of the right and left tibias (i.e., tibia-to-tibia spike delay) was computed. This served as a verification procedure to confirm that the values were consistent with the expected step times and that no anomalies were present, which would indicate errors in spike selection.

#### 2.2.2. Kinematic–Temporal Analysis

A ground-level, rear-view high-speed camera (Mars, 800 fps, Contrastech, Hangzhou, China) was used for photogrammetric analysis to determine flight and contact times (Figure 1). The software used for recording was iCentral 2.3.5 (Contrastech, Hangzhou, China). To determine the contact and flight times, the videos were analyzed in the Kinovea 0.9.5 software, where the moments of landing and take-off of the foot on the ground were manually determined (obtaining a total of 20 steps per sector). The events were exported to an Excel template, which allows for calculating the flight and contact times of each foot (this template is provided as Supplementary Materials). Cadence was calculated based on the acceleration data, determining the number of sacral spikes per minute [37]. For descriptive purposes, to compare with the accelerometer variable, the step time was computed for each runner by summing the contact and flight times. Also, for descriptive reasons and based on the visual analysis of videos recorded using a ground-level, rear-view high-speed camera, runners’ foot strike patterns were categorized as rearfoot, midfoot, or forefoot. This classification was performed by two experienced investigators, who reached a consensus on each runner’s foot strike pattern.

### 2.3. Data and Statistical Analysis

For the statistical analysis, acceleration spikes’ magnitude and attenuation were averaged for each minute of the 30 min fatigue running test. To calculate the symmetry index as a percentage, the difference between the values of the right leg and the left leg steps was calculated per minute, divided by the mean of both values, and the result multiplied by 100 [12]. This calculation was applied to acceleration spike magnitude and attenuation and for the contact time. A positive value indicates a higher value in the right tibia and vice versa.

The statistical analysis was performed with OriginLab 2019b software, setting the significant *p* value at *p* = 0.05. Descriptive data are presented as the mean ± standard deviation. Cardiovascular and body composition parameters were compared between the two days via a paired *t*-test, to ensure that there were no changes. As a control measure, the relationship between the step time computed via photogrammetry and the time between consecutive tibia spikes (which is supposed to be near the step time) was studied using a linear regression.

To study the relationship between fatigue, asymmetries, and acceleration spike variables, data from all runners were averaged per minute throughout the fatigue test and a linear regression model was computed, considering the minute of the running test as the independent variable. To evaluate the effect size of the correlations, the scale from Evans [38] was used. It establishes five qualitative levels: (1) 0.00–0.19, very weak; (2) 0.20–0.39, weak; (3) 0.40–0.59, moderate; (4) 0.60–0.79, strong, and (5) 0.80–1.0, very strong. The coefficient of determination (r^2^) was also included as its interpretation offers certain advantages over Pearson’s r. Specifically, the coefficient of determination represents the shared variance between two variables and allows for direct comparison as a ratio, whereas correlation coefficients do not [39].

To compare the differences between sectors in the kinematic–temporal variables (contact, flight times, and cadence), a repeated-measures ANOVA test was performed. Sphericity was considered, and if the Mauchly test yielded a *p* value less than 0.05, the Greenhouse–Geisser correction was applied. In this case, the effect size was calculated using the partial eta squared (η^2^_p_) by dividing the sum of squares of the intercept by the sum of the sum of squares of the intercept plus the sum of squares of the error. The effect size was interpreted as small (0.01), medium (0.09), or large (0.25) [40].

## 3. Results

**Figure 2:**
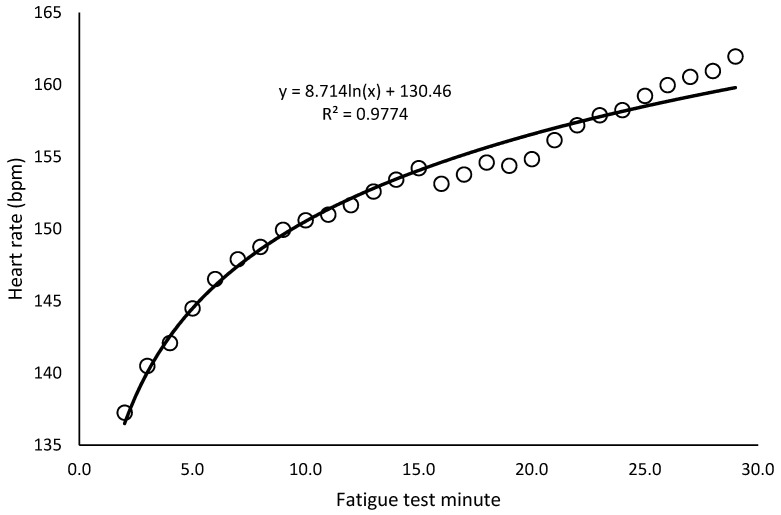
Heart rate trend in the 30 min constant-speed fatigue test (average of all runners).

Temperature and humidity ranged between 22 and 25 degrees Celsius and 30–40%, respectively. The paired *t*-test showed no significant differences in any body composition or cardiovascular variable between the two days (*p* > 0.05 in all cases). In the incremental test, the minimum and maximum speeds reached were 13 and 20 km/h (16.4 ± 1.5 km/h). In the fatigue test, the minimum speed selected based on the incremental test was 10.4 km/h and the maximum speed was 16 km/h (13.1 ± 1.2 km/h), and heart rate increased following an approximately logarithmic trend (r = 0.99; *p* < 0.001) (Figure 2). Of the 18 runners, only 1 was unable to complete the test due to exhaustion approximately in the 20th minute. The portion of the test completed by this participant was included in the analysis.

**Figure 3:**
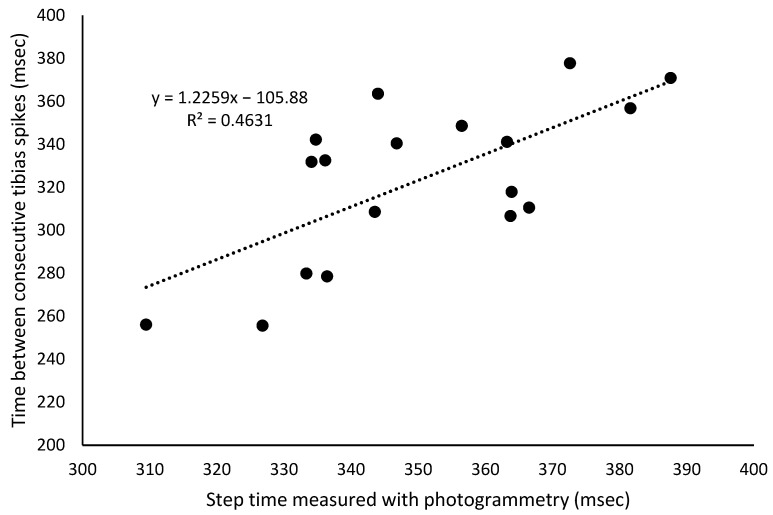
Linear regression line and equation of the relationship between the step time measured with photogrammetry and the time between consecutive tibia spikes.

Tibia-to-sacrum spike delay (msec) showed a mean difference of approximately 65 msec for the right and left steps, which increased throughout the test (r = 0.96; *p* < 0.001). However, the rate of increase was less than 0.7 msec per minute for both legs. The tibia-to-tibia spike delay (msec) had a mean value of 323 ± 37 msec. There was a strong relationship between the step time measured with photogrammetry and the time between consecutive tibia spikes (Figure 3), computed with the accelerometers, providing a measure of the internal validity of the accelerometer data.

Regarding foot strike patterns, only one of the eighteen runners exhibited a forefoot strike pattern, while three displayed a midfoot strike pattern and fourteen had a rearfoot strike pattern.

### 3.1. Asymmetries’ and Acceleration Spikes’ Trend in the Fatigue Test

**Figure 4:**
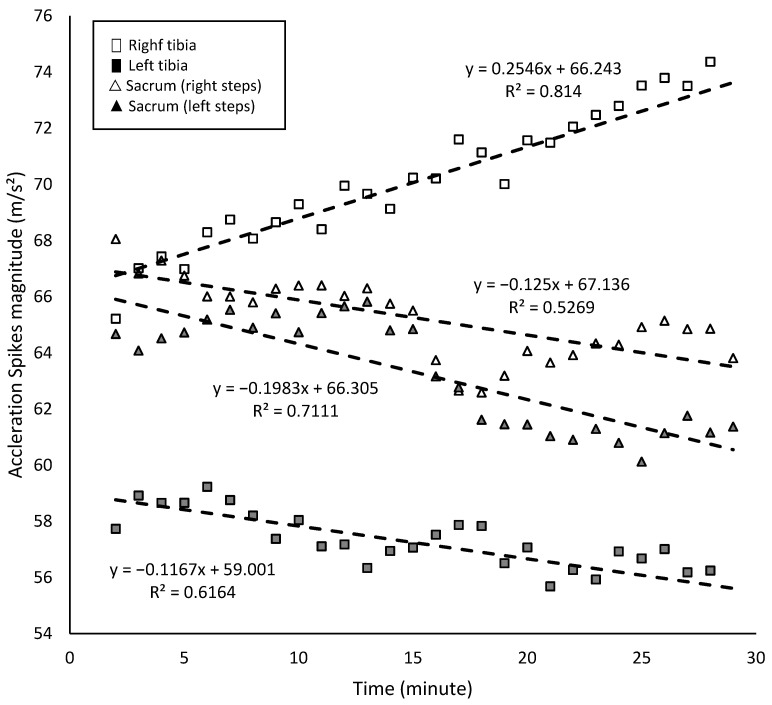
Acceleration spikes in tibias and the sacrum region (m/s^2^) in the 30 min fatigue test.

During the running test, there was a significant increase in the acceleration spikes’ magnitude for the right tibia (r = 0.9; r^2^ = 0.81; *p* < 0.001) and a significant decrease in the acceleration spikes’ magnitude for the left tibia (r = −0.79; r^2^ = 0.62; *p* < 0.001) (Figure 4). A linear regression model indicated that, for each minute of the fatigue test, acceleration spikes in the right tibia increased by approximately 0.25 m/s^2^, whereas those in the left tibia decreased by 0.10 m/s^2^ (Figure 4). In the sacral region, the magnitude of acceleration spikes decreased for both right (r = −0.73; r^2^ = 0.53; *p* < 0.001) and left steps (r = −0.84; r^2^ = 0.71; *p* < 0.001), with reductions of approximately 0.13 m/s^2^ and 0.20 m/s^2^ per minute, respectively (Figure 4). In addition, with fatigue, the attenuation of acceleration spikes in the right tibia steps increased significantly (r = 0.89; r^2^ = 0.79; *p* < 0.001), but this was not the case for the left tibia steps (r = −0.26; r^2^ = 0.06; *p* = 0.173).

**Figure 5:**
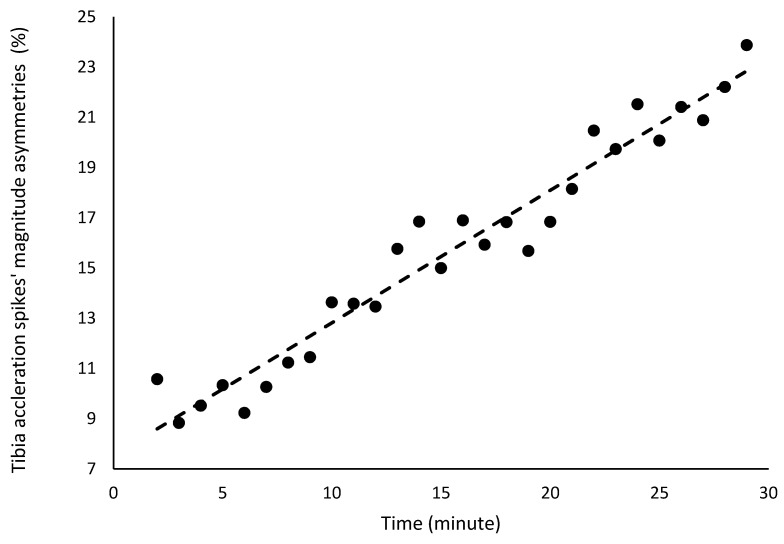
Acceleration spikes’ asymmetries in the tibias and sacrum region in the 30 min fatigue test.

There were no significant changes in the asymmetries of sacral acceleration spike asymmetries or attenuation (Table 2). However, a significant increase was detected in the asymmetry of tibial acceleration spike magnitude, which rose by 0.5% per minute, ranging from approximately 9% at the beginning of the test to 25% at the end (Table 2; Figure 5). The coefficient of determination indicates that 95% of the variance in the asymmetry of tibial acceleration spike magnitude was explained by the minute of the fatigue test.

### 3.2. Kinematic Differences Between Sectors in the Fatigue Test

**Figure 6:**
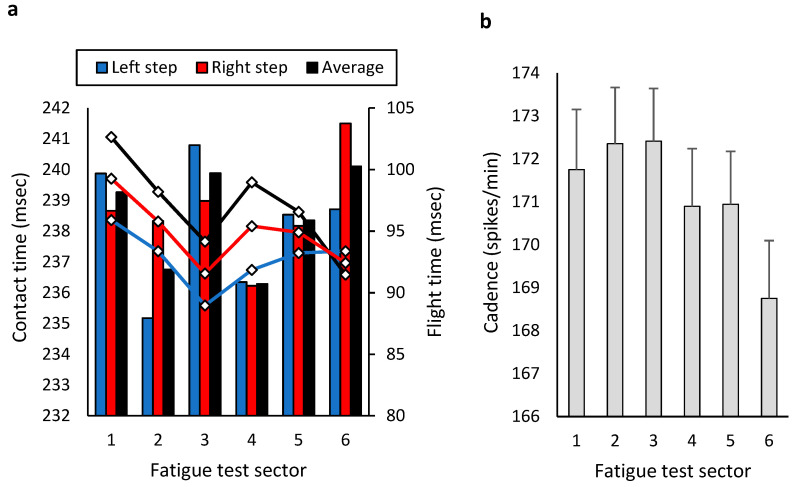
Kinematic–temporal data from each 5 min sector of the 30 min fatigue test: (**a**) contact and flight times and (**b**) cadence.

During the fatigue test, no significant differences were observed between sectors in contact times (F = 1.014; *p* = 0.334; η^2^_p_ = 0.06), flight times (F = 1.295; *p* = 0.277; η^2^_p_ = 0.07) (Figure 6a), cadence (F = 2.252; *p* = 0.128; η^2^_p_ = 0.12) (Figure 6b), or contact times’ asymmetries (F = 0.860; *p* = 0.511; η^2^_p_ = 0.05).

## 4. Discussion

The aim of this study was to analyze the effects of fatigue generated by a 30 min continuous running test at moderate speed on the lower limb acceleration spike asymmetries, on the acceleration spikes themselves, and on kinematic–temporal variables. The fatigue test induced significant differences between sectors in acceleration spike asymmetries but did not affect kinematic–temporal variables. Specifically, there was an increase in the right tibia acceleration spikes, a decrease in the acceleration spikes of the left tibia, and consequently, an increase in the acceleration spikes’ asymmetries (Figure 3). These results suggest that fatigue induces a deterioration in running mechanics, as evidenced by greater asymmetries due to higher acceleration spikes on the right (dominant) limb. This is not consistent with previous literature findings and needs to be further studied.

The magnitude of acceleration spikes tended to increase, similar to the findings by other authors [9,27]. However, this trend was only observed in right steps, suggesting that the ability to absorb acceleration spikes in the right limb decreases with fatigue. One possible explanation is that ankle joint stiffness diminishes over time, reducing its capacity to absorb impact peaks [7,10,11,41]. Sanno et al. [42] stated that this loss of ankle stiffness will increase the acceleration spikes in the tibial area. Additionally, fatigue may alter movement patterns and muscle activation, for example, by challenging agonist–antagonist coactivation mechanisms in the load response phase or increasing the load on the passive elements (joints and bones) [9,43]. In contrast, acceleration spikes in the left tibia decreased over time, possibly due to a compensatory technical modification to protect the weaker, non-dominant leg. The present study also found a positive trend in the attenuation index for right steps, supporting the hypothesis that running technique is adjusted with fatigue to minimize high-impact forces reaching the upper body [44]. This trend was not observed in left steps, possibly because the lower acceleration magnitude did not require a similar movement pattern adjustment. Some authors report a deterioration of attenuation as fatigue increases [30], while others find an increase in attenuation [44]. Moreover, these apparent contradictions may have been the result of methodological differences between studies such as running surface, fatigue protocol [29], or running mechanics between subjects [25,45]. Related to this latter point, each runner may change their motor pattern differently to mitigate the negative effects of fatigue, depending on their anatomy, morphology, physiology, or level of training [45].

Regarding kinematic–temporal variables, our hypothesis that fatigue would induce changes was not fulfilled. However, there are conflicting data in the scientific literature. For example, Möhler et al. [10] in a study on middle-distance racing or Garcia-Pinillos et al. [7] during a 60 min treadmill run found an increase in contact time and a decrease in flight time with fatigue, reflecting neuromuscular deterioration associated with fatigue, but no changes in step length or cadence. Otherwise, Mizrahi et al. [9] in a 30 min run reported a decrease in cadence, which is indicative of a less metabolically efficient run. Hunter and Smith [8] suggested that cadence changes in a 60 min run were subject-specific, with some runners exhibiting little to no change. Additionally, other authors did not find changes with fatigue in most kinematic–spatiotemporal parameters, including contact times [22]. Hanley and Tucker [22] found differences in flight time, but only after the fifth kilometer of the running test. Therefore, the absence of significant differences in temporal variables in the present study is not unexpected, as several studies have also failed to find them. Nevertheless, the kinematics and the acceleration data are supposed to be interrelated; hence it is reasonable to assume that the reported acceleration spike changes translate into changes in flight, contact times, or cadence. It is possible that the kinematic alterations in a continuous 30 min test at the selected speed are negligible and that very sensitive biomechanical measurement systems are required to detect them. In our study, an 800 fps camera was used to determine contact and flight times, which we considered to be quite sensitive. However, it may not have been precise enough to capture subtle variations. Additionally, the magnitude of kinetic changes in recreational runners with an adequate level of training may not be large enough to produce detectable kinematic alterations.

Studies analyzing the effect of fatigue on kinetic or kinematic asymmetries are relatively recent, and their conclusions remain inconsistent. While a few report that fatigue does increase asymmetries, mainly in kinematic variables [13,15,24], others state that it does not [3,13,14,16,17,18,19,20,21,22,23]. Some research even states that fatigue decreases asymmetries, which may indicate a protective strategy in which loads are more evenly distributed between limbs as fatigue progresses [25]. Buxton et al. [17] observed that while no significant differences were found at the cohort level, individual analyses revealed variations, with some subjects exhibiting increased asymmetries as fatigue progressed. The main finding of our study is that fatigue, generated by moderate-speed running, increased asymmetries in the acceleration spikes between the right and left legs. This finding is different from most previous studies, which found no change in kinetics’ asymmetries with running fatigue. Our results are consistent, however, with Gao et al. [24], who found that under fatigue, the knee maximum extension velocity was greater in the dominant limb, which may be due to the greater propelling contribution of this leg in locomotion [2]. A higher knee extension velocity would imply a more aggressive impact of the foot against the ground and thus a higher acceleration spike. Additionally, Radzack et al. [13] found that the decrease in stiffness was less apparent in the right limb, which would also generate higher acceleration spikes (greater stiffness implies less deformation of the ankle, which would result in a higher impact, by Newton’s third law). This is contrary to the thesis of Sanno et al. [42], who speculate that stiffness and acceleration spikes are positively related.

The increase in acceleration spike asymmetries with fatigue could be attributed to a combination of two well-documented factors: (1) fatigue-induced impairments in running mechanics and (2) inherent structural and functional differences between the lower limbs. Concerning the first point, fatigue is supposed to cause impairments in neuromechanical factors, such as a reduced stretch reflex sensitivity and muscle stiffness and a deterioration in the force potentiation mechanisms [46]. As for the second factor, structural and functional differences between the limbs are well-established [1]. The dominant leg (typically the right) exhibits greater propulsive capacity [2] and an increased ability to develop force in the extensor muscles [47], while the non-dominant leg may be a little larger [1], with a better stabilization or braking capacity [2] and an increased ability to develop force in the flexor muscles. Given these disparities, it is unlikely that fatigue affects both legs equally. One plausible explanation is that the right leg, being more engaged in propulsion, experiences greater fatigue-induced deterioration in its impact attenuation mechanisms. This would result in an increased mechanical load, as evidenced by the rise in acceleration spikes in the right tibia. Concomitantly, the mechanical loading of the non-dominant leg may decrease due to a reduction in its involvement in the braking phase of the stance, induced by fatigue (the acceleration spike on the left leg decreased significantly with time). However, these hypotheses cannot be answered with the methodology employed as no force plates and/or electromyography were used. Consequently, it is not possible to determine the precise contribution of each leg during different phases of the running cycle. Maybe the differences with other studies that found no asymmetry changes with fatigue could be due to the instrumentation and the variables measured, i.e., methodological issues. The present study measured the acceleration spikes in every single step during the whole run and not only in a few cycles.

This study has several limitations that should be considered when interpreting the findings. Firstly, physiological thresholds were not measured, which meant the nature of the test (aerobic vs. anaerobic) could not be fully determined. Nevertheless, the heart rate increased progressively, indicating that the pace was close to the anaerobic threshold (otherwise, the heart rate would have maintained a quasi-steady state). Secondly, the level of the runners included was not considered for the statistical analysis and the sample was somewhat heterogeneous. In this regard, Strohrmann et al. [34] indicated that beginner and intermediate runners attenuated less shock with fatigue and that they had a greater increase in acceleration spikes with fatigue. This suggests that future research should include larger sample sizes and categorize participants based on their running experience to better understand how expertise influences fatigue-related asymmetries. Additionally, while some studies suggest that accelerometers may not be suitable for measuring mechanical loading [48,49,50], a substantial body of literature supports their use for this purpose [9,51,52], with studies also indicating that accelerometers are valid tools for estimating ground reaction forces [53,54]. Finally, ground reaction forces, joint stiffness, and muscular electrical activity were not measured (or other local measurements of fatigue), nor was the acceleration signal evaluated in the frequency domain. These measures could provide deeper insight into the observed increase in mechanical load and asymmetries. Despite these limitations, our work should be taken into account, considering that it is one of the scarce studies that find an increase in asymmetries in acceleration spike variables with fatigue and that these variables are proposed to be associated with overuse injury and running efficiency. Future studies should aim to measure these variables continuously and integrate acceleration data (both in the time and frequency domains) with force plate assessments, kinematic parameters, and muscle activity measurements (e.g., electromyography and joint stiffness). To analyze the effects of fatigue on asymmetries in acceleration spikes in a more realistic context, it would also be valuable to investigate the effects of fatigue during training sessions in overground running, where speed is not constant and is self-regulated by each runner. Also, it has been suggested that although the parameters estimated during treadmill running are comparable to those measured during overground running, they are not equivalent [55]. Additionally, exploring the relationship between asymmetries in acceleration spikes and metabolic cost should be of particular interest, as some studies have found a correlation between these variables [6] while others have not [56]. Finally, the results highlight the importance of measuring the kinematics and kinetics of both lower limbs and not just of the right (dominant) leg, especially when running fatigated, even at moderate speeds.

## 5. Conclusions

Few studies analyze the effects of fatigue on asymmetries, despite their potential implications for running efficiency and injury risk. The results of this research point out that, in a 30 min continuous test at moderate speed, asymmetries in acceleration spikes undergo notable changes. Specifically, there was an increase in tibial acceleration spikes in the right leg and a decrease in the left leg, leading to greater asymmetries between the limbs. These findings suggest that fatigue alters the biomechanics of the right (dominant) and left (non-dominant) legs differently, probably due to local neuromechanical or physiological factors, which should be investigated in depth. Future research should combine continuous data collection methods such as accelerometers with force platforms and parameters related to local muscular fatigue. Also, it is suggested that fatigue-focused studies should collect data from both lower limbs and not just the right (dominant) one.

## Figures and Tables

**Table 1:** Anthropometric and cardiovascular data of the subjects.

Parameter	Minimum	Maximum	Mean	Std.
Age (years)	22.2	49.6	35.6	7.5
Height [cm]	153	190	172.8	9.0
Weight [kg]	47.3	88.6	68.9	11.2
BMI [kg/m^2^]	20.2	27.6	22.9	2.1
FM [%]	8.3	22.8	14.7	4.1
FFM [kg]	36.6	72.8	55.9	9.5
Body water [%]	55.6	67.2	62.0	3.4
Resting heart rate [lpm]	53	84	63.2	8.5
Systolic blood pressure [mmHg]	91	170	119.9	16.4
Diastolic blood pressure [mmHg]	61	92	71.7	8.8
Running experience (years)	5	25	12.3	4.6
Running distance per week (km)	10	100	31.5	23.8

BMI: Body mass index; FM: Fat mass; FFM: Free fat mass.

**Table 2:** Regression line equations for the variables used to measure acceleration spike asymmetries (independent variable: minute during the 30 min fatigue test).

Asymmetry (%)	Mean ± SD	Pearson’s r	Slope	Intercept	t	*p* Value
Tibia acceleration spikes (%)	16 ± 5	0.98	0.53	7.5	25.59	<0.001
Sacrum acceleration spikes (%)	4 ± 2	0.34	0.07	2.9	1.88	0.0714
Attenuation (%)	−15 ± 281	−0.02	−0.81	−2.1	−0.1	0.7291

SD: Standard deviation.

## Data Availability

Data are unavailable due to privacy or ethical restrictions. If any researcher is interested in the raw data of this study, please write to the corresponding author.

## Supplementary Materials

The following supporting information can be downloaded at https://www.mdpi.com/article/10.3390/bioengineering12030294/s1, Supplementary file S1: Template used to compute contact times and flight times, Supplementary file S2: Template used to select adjacent peaks.
